# The impact of the war on maintenance of long-term therapies in Ukraine

**DOI:** 10.3389/fphar.2022.1024046

**Published:** 2022-11-24

**Authors:** Nataliia Khanyk, Bohdan Hromovyk, Oksana Levytska, Tamas Agh, Björn Wettermark, Przemyslaw Kardas

**Affiliations:** ^1^ Uppsala Universitet, Uppsala, Sweden; ^2^ Danylo Halytsky Lviv, National Medical University, Lviv, Ukraine; ^3^ Syreon Research Institute, Budapest, Hungary; ^4^ Department of Family Medicine, Medication Adherence Research Centre, Medical University of Lodz, Lodz, Poland

**Keywords:** long-term therapy, war, Ukraine, medicines, healthcare system, armed conflicts (MeSH), disaster pharmacy, disaster medicine

## Abstract

Due to the Russian invasion, which started on 24 February 2022, the Ukrainian healthcare system is facing multiple challenges. A great number of healthcare facilities have been destroyed, while availability of other ones is often limited due to a lack of qualified medical staff. Certain services, e.g. cancer therapies, have been seriously disrupted. Moreover, millions of Ukrainians with chronic conditions are also suffering as due to war-related problems with execution of their long-term therapies. Availability of drugs is particularly limited in the occupied regions. According to the national statistics, as of 18 August 2022, about 505 pharmacies were damaged in Eastern Ukraine and 47 completely ruined. Moreover, the invaders have been blocking humanitarian aid provided to these territories by the Ukrainian government or other countries. Fortunately, in the areas controlled by the Government of Ukraine, the acute shortage of medicines, observed at the beginning of the war, has already been eliminated. Nevertheless, not all drugs are now fully available, even in the areas where no military attacks occur. The economic availability of drugs is also profoundly influenced by the significant increase in the cost of medications and the fall in average salaries. The Government of Ukraine is trying to minimise the impact of these war-related challenges by adopting a new legislation. This includes, among others, simplification of procedures for licensing, quality control and import of medicinal products to Ukraine. Other measures involve securing displaced people with the option of benefiting from local healthcare facilities, broadening the scope of the ePrescription system, authorizing primary care doctors to issue prescriptions to refugees, increasing the number of drugs reimbursed for long-term therapies, *etc.* These solutions, however, cannot balance all the harmful consequences the war in Ukraine brings in terms of maintenance of long-term therapies. Therefore, in order to minimise this negative impact, Ukraine still needs urgent international support in this area.

## Introduction

**FIGURE 1:**
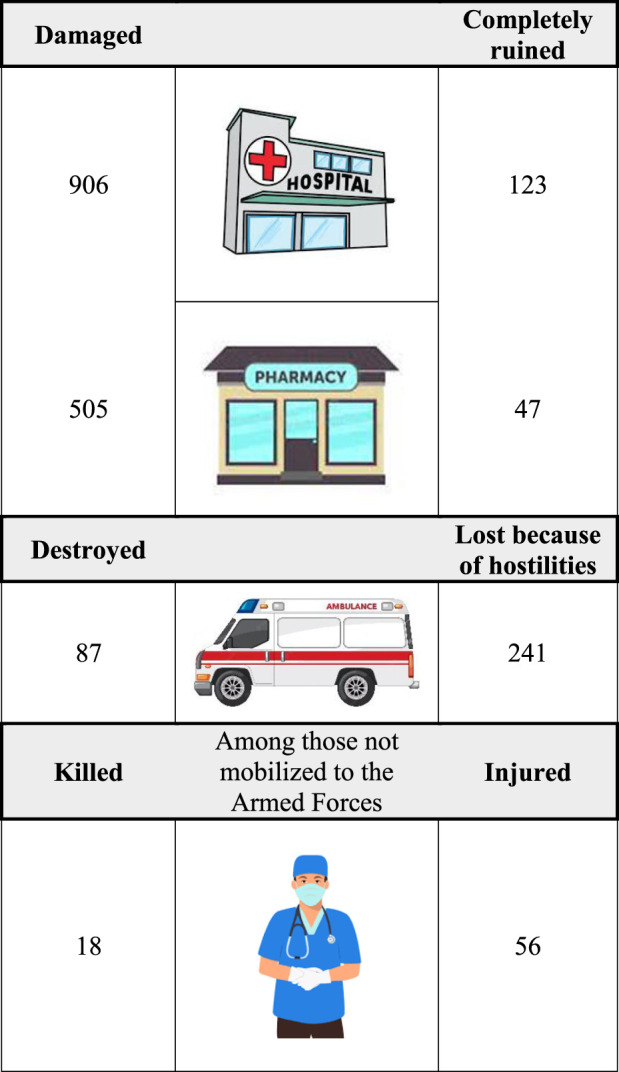
Statistics of war-related losses of the Ukrainian healthcare system, as of August 2022 - preliminary data due to a lack of access to some parts of the territory (Skrypnyk, 2022d).

Russia launched a full-scale attack on Ukraine on 24 February 2022, invading many places in the East, South (the coast), West, North as well as in the central (national capital Kyiv) region of the country. The war continues and exerts a major impact on the entire national healthcare system. In the first 7 months of the war, 906 health care institutions were seriously damaged and 123 were completely ruined, at least part of them intentionally (Skrypnyk, 2022d). Moreover, 87 ambulances were destroyed and another 241 medical cars were lost as a result of hostilities (Skrypnyk, 2022d; kapri, 2022) (see Figure 1). These are just preliminary data due to a lack of access to the occupied territories, yet the analysis of the situation in the de-occupied territory showed that virtually every healthcare institution located there was damaged or destroyed (Skrypnyk, 2022c). At least 18 healthcare workers from among those who were not mobilized to the Armed Forces (Skrypnyk, 2022c) were killed and more than 56 were injured (Skrypnyk, 2022d), (glavcom, 2022). The occupied territories face a dramatic shortage of healthcare professionals. To give an example, in Melitopol, which has 150,000 inhabitants, 50% of doctors left in the first months of the war. Those who stayed are able to provide emergency care only and cannot guarantee the citizens maintenance of long-term therapies (Skrypnyk, 2022a).

The consequences of this scenario are more than profound. A recent analysis indicates that a war-related delay in care for only 4 months for five prevalent types of cancer will lead to an excess of over 3,600 cancer deaths in the Ukrainian population in the subsequent years (Caglevic et al., 2022). However, it is not only the management of life-threatening conditions that is seriously affected by abnormal circumstances resulting from the current military conflict. With its population exceeding 40 million, Ukraine has millions of patients who require long-term therapies for hypertension, diabetes, asthma, COPD and numerous other chronic conditions, which due to their high prevalence, are of the greatest importance to public health. The World Health Organisation predicts that disruption of these therapies will bring negative consequences, i.e. increased morbidity and mortality, which altogether constitute another detrimental effect of the war (reliefweb, 2022).

The armed conflict affects the maintenance of long-term therapies in many ways. Millions of Ukrainians were forced to leave their homes and move to other locations - some of them within the territory of their country, while others much farther, abroad. As a result, their access to the healthcare system was severely restricted, which made availability of their chronic medications very challenging. Others, who stayed in their country, are often deprived of access to medications due to military operations in the area. Even in the case of those lucky ones who live in safer locations, such as Western Ukraine, access to drugs is seriously limited because of reduced production, broken chains of distribution, and last but not least, rapidly rising prices. This paper describes these challenges in more detail, providing a snapshot of the scenario as of early August, 2022 (unless otherwise stated). It also presents various actions that Ukraine has been taking to minimise the effect of these challenges, and to ensure continuation of treatment to patients who require long-term therapies, despite the existing unfavourable conditions.

### Impact of the war and related shortage of medicines

The war in Ukraine created a serious barrier to access to pharmaceuticals which constitute the core element of long-term management in non-communicable diseases. The acute shortage of medicines at the beginning of the war posed a major challenge to maintenance of long-term therapies in Ukraine. Fortunately, this shortage is no longer so serious in the territory controlled by the Government of Ukraine (Kupriianova, 2022: Ternova, 2022a), as the pharmaceutical sector gradually resumed its work. Additionally, the drugs transported to Ukraine as humanitarian aid are delivered to hospitals or dispensed at mobile points (Sherfetdinova-Sushchyk Zebede. Ministry of Health, 2022).

However, in the occupied territories the situation is uncontrolled and thus much worse since it is not possible to provide medications to people. For example, the Kherson region, currently occupied by the Russian invaders, faces a crisis resulting from shortage of medical supplies. There are even problems with food delivery. Drugs used in the treatment of chronic diseases (especially oncologic medications) are practically not available to people. In absence of family doctors caused by the war, only clinical hospitals operate in Kherson and in some of them, entire departments ceased to function as most of the highly qualified medical workers left the occupied territories. Those who stayed are overstrained with work and feel constant moral pressure from the occupation authorities. Moreover, the occupiers decided to cancel previously introduced reforms in the Ukrainian healthcare system, including reimbursement of medicines (Skrypnyk, 2022b; Return to the past, 2022). The same problem exists in the occupied Mariupol where people are dying due to lack of medications. There is a shortage of medicines for cancer, diabetes, tuberculosis and thyroid problems (Skrypnyk, 2022b). The Russian invaders are blocking humanitarian aid provided to these territories by the Ukrainian government or other countries. Only individual volunteers, using their own means of transport, are trying to distribute necessary goods, including medications, to the people. Due to very limited travelling options, in Kherson those who want to flee the occupied area have to wait in long queues for being transported to safe places. They are often exposed to difficult weather conditions, without any chance for medical support, risking death resulting not only from military operations but also from their indirect short-term consequences, such as hunger, infections, *etc.*

Unfortunately, it is not the end of the list of health repercussions brought by hostilities. When it comes to chronic conditions, lessons learned from previous armed conflicts show that, in the case of Ukrainian people, other sequelae may also occur. These include a variety of stress-mediated conditions, such as exacerbations of cardiovascular diseases, strokes and heart attacks, cancers and many more, which, in turn, may lead to substantially increased mortality among civilians, including children who are burdened most severely (Jawad et al., 2020; Sadetzki et al., 2017; Al-Makhamreh et al., 2021).

### Effects of the economic crisis on availability of medicines

The war led to a severe decline in the Ukrainian economy, causing a significant increase in the budget deficit, and critical dependence of the Ukraine’s economy on international aid (Kirsanov, 2022b). In June/July 2022, more than 50% of the deficit was covered by financial assistance from international partners (Zanuda). However, the International Monetary Fund estimates that the GDP decline may reach the level of 33–35% by the end the year and predicts a significant increase in public debt to more than 85% of the GDP (Kirsanov, 2022b). Moreover, Ukraine is facing high rates of inflation. The National Bank of Ukraine predicts that by the end of the year prices may rise by at least 30% (Zanuda). Research conducted in Ukrainian pharmacies showed a 20–25% increase in prices (Murashko, 2022), and in some of them it was up to 60% (Kupriianova, 2022; Murashko, 2022). The experts predict even further increase. It is noteworthy that in the same time, salaries in private sector companies and enterprises decreased by 10%–50% as compared to the pre-war period (Department of Monetary Policy, 2022; Sherfetdinova-Sushchyk Zebede. Ministry of Health, 2022). All these factors have profound consequences for maintenance of long-term therapies, as higher costs of medicines reduce their affordability.

The factors that play the main role in the rapid increase in drug prices are, among others, more difficult logistics due to current shutting off of air and sea transport routes, as well as destruction of many warehouses storing medications and raw materials by the Russian invaders. Unlike in the pre-war period, delivery of these raw materials now takes months or more (Ternova, 2022b). It should be emphasized that up to 75% of the drugs distributed in Ukrainian pharmacies are produced locally. However, almost all raw materials for drug manufacturing, which have become more expensive all over the world, are imported (Kupriianova, 2022). Another problem is a higher USD to UKH (Hryvna, Ukrainian currency) exchange rate, as well as growing prices and shortage of fuel.

Consequently, since March 2022 sales in the Ukrainian pharmaceutical market have dropped significantly - by 11% in March, by 32% in April and by 24% in May and June as compared to the same period of 2021. The reasons for sales decline are directly related to the war, i.e. massive migration from the country, the occupation of specific Ukrainian regions, the considerable medical humanitarian aid provided by other countries, and drop in the income of the population that began to choose less expensive drug analogues to save on medications (Kirsanov, 2022a). Interestingly, the downward trend began to develop just after a substantial growth of the Ukrainian pharmaceutical market observed before the beginning of the war (by 31% in January and 45% in February). In fact, the volume of pharmacy sales was doubled in the first 11 days of the Russian invasion as a result of large amounts of medications purchased by patients with chronic conditions. The top most often bought medicines represented the following ATC classes: M01 - antiinflammatory and antirheumatic products, C09 - agents acting on the renin-angiotensin system, N02 - analgesics, N06 - psychoanaleptics, A10 - drugs used in diabetes, J01 - antibacterials for systemic use (Kirsanov, 2022a).

### Decrease in the physical availability of medicines

The decrease in the physical availability of medicines during the war was related to reduction in the number of working pharmacies and destruction of their infrastructure. Only around 10% out of 22,780 Ukrainian pharmacies continued to work in the first days of the war (interfax, 2022b). However, at the beginning of April 2022, the share of working pharmacies increased to 71% (apteka, 2022a). According to national statistics, as for 18 August 2022 505 pharmacies were damaged in eastern Ukraine and 47 completely ruined (Skrypnyk, 2022d), whereas at least 112 pharmacies located in the areas affected by hostilities were either not able to work (Sherfetdinova-Sushchyk Zebede. Ministry of Health, 2022) or were captured by the invaders. The effect of this scenario in large cities was not so dramatic. It should be remembered that the number of pharmacies per capita in Ukraine prior to the war was 2.5 times higher than, for example, in Germany. Nevertheless, shutting down of scarce pharmacies operating in rural areas created serious problems with the local availability of medicines (Ternova, 2022b).

Military operations in certain regions also made transport of medicines difficult or even impossible. Warehouses with medicines in these zones were blocked, which influenced the logistics of pharmaceuticals in the whole country (Nynko, 2022). For example, warehouses with ready-made medicines and raw materials of one of the biggest Ukrainian pharmaceutical producer, JSC “Farmak” (Kyiv), were burned by the occupiers, which made the company suffer a UAH 1.5 billion loss (interfax, 2022a).

Additionally, there was a shortage of pharmaceutical sector staff due to high migration of the population, as well as their active involvement in defending Ukraine against the Russian army. At the beginning of October, a shortage of pharmacists may still be observed in the regions close to the war zone [38]. In particular, there is a lack of personnel in the pharmaceutical industry, such as specialists with high qualifications (Ternova, 2022b). In order to address the issue, students and graduates of pharmaceutical and medical educational institutions who have not yet completed an internship were allowed to work in pharmacies (studentam-medichnih, 2022).

### Changes in legislation adopted to overcome current problems

Ukraine tries to flexibly adopt its legal and normative frameworks to the extraordinary war scenario in an attempt to overcome the difficulties. Thus, in the first month of the war, the government body adopted 29 orders that related to various aspects of medical and pharmaceutical services for all population categories toward Russian military aggression (Zhdan et al., 2022). It also refers to the maintenance of long-term therapies. One of these steps is sim*plification of the procedures for licensing, quality control and import of medicinal products* to Ukraine, which provides an option of emergency state registration of medicinal products by simplifying requirements with regard to labelling and expiration dates of imported medicines (drlz, 2022; Procopenko, 2022). Before the war, imported medicines should have the expiration dates of at least half of the period specified by the manufacturer. According to the new changes this period is not limited but should not be expired. Drugs which are already registered in Ukraine can be imported to its territory now without labelling in Ukrainian language, provided that they accompanied by instructions for use (patient leaflet) approved in Ukraine, and a warranty letter. Medicinal products which are not registered in Ukraine can be imported only for the provision of the Armed Forces of Ukraine and health care institutions (except pharmacies), without right for retail sale.

Drug reimbursement is a new issue in Ukraine as it was initiated as late as in 2017. It applies to the outpatient treatment of selected conditions only, i.e., cardiovascular diseases, bronchial asthma, diabetes, mental and behavioural disorders, and epilepsy. It covers only 368 various medicines (116 free of charge) and 76 insulin preparations (47 free of charge) and some changes in their quantities are expected. The program continues to work in the territory controlled by the Government of Ukraine with several changes made to the procedure of its implementation. Medicines subject to reimbursement can be prescribed and dispensed on electronic or traditional paper prescriptions by any general practitioner regardless of the patient’s place of residence, unlike in the pre-war conditions. Nevertheless, the number of prescriptions has decreased. Not all trade names of reimbursed medicines are available now. The National Health Service of Ukraine pays back the money to the pharmacies for the dispensed reimbursed drugs. However, not all pharmacies which participated in this program before the war continue to dispense reimbursed medicines. Thus, patients are recommended to make sure whether a specific pharmacy provides the reimbursement option in advance (Affordable Medicines, 2022).

According to the national statistics, prior to the war (i.e. as of 1 February 2022) there were 41,130,400 Ukrainian inhabitants (index.minfin, 2022). Since the beginning of the full-scale war, almost nine million citizens have been displaced across the territory of Ukraine and beyond its borders. Almost six million citizens are registered abroad, and almost four million have been awarded the temporary protected status in their host countries. As many as 90% of refugees are women and children. According to the UN Refugee Agency, hundreds of thousands of Ukrainians have been forcibly deported to the territory of the invader state. The National Information Bureau has already identified more than 5,600 children deported to the country of the aggressor (varta, 2022; minre.gov, 2022).

The increased amount of work that both medical staff and the whole healthcare system in general has to face was observed in regions with higher migration of people due to a change in the structure of the population. To overcome these difficulties, new changes in legislation were adopted. All internally displaced people who moved or were forced to change their place of residence can apply for primary medical help to any health care institution of their choice. It includes emergencies, primary medical care and vaccinations in accordance with the Preventive Vaccination Calendar. Records keeping of such patients is carried out. According to the law, doctors are obliged to issue prescriptions for necessary drugs, including medications covered by the reimbursement program, using e-Prescriptions or, in case of no access to the electronic healthcare system, paper prescriptions (ombudsman, 2022).

The Ministry of Health of Ukraine continues to gradually introduce the electronic prescription scheme. From 1 August 2022 a new functionality of e-Prescription for antibiotics have been introduced in Ukraine. Of a note is that before these changes, antibiotics could be bought from Ukrainian pharmacies without any prescription. Now, only pharmacies located on the frontline or under occupation will be able to dispense antibiotics to patients without medical doctor’s prescriptions (Electronic prescription for antibiotics, 2022). As an exception, voluntary and charitable organizations can purchase antibiotics directly from distributors without prescriptions at the request of relevant institutions, military units or healthcare organizations. The next stage will be introduction of e-Prescription for narcotics, and the final stage will be the application of the e-Prescription system for all prescription drugs (Horbunova, 2022; Radutsky, 2022). There was no frantic demand for antibiotics before the introduction of e-Prescriptions as compared to the chaotic supply of these medications at the beginning of the COVID-19 pandemic and in the first weeks of the war (Electronic prescription for antibiotics, 2022).

The new amendments to the Ukrainian Law on Medicinal Products sets forth that the state registration of medicines may be refused or cancelled by terminating or shortening the validity period of the registration certificate if one, several or all stages of the production of the medicinal product are carried out by enterprises whose production facilities are located in the territory of the Russian Federation or the Republic of Belarus. A similar scenario may occur if an owner of the registration certificate or their representative has any kind of relations with business entities in the territory of above-mentioned countries (biz.ligazakon, 2022). However, having in mind that a high proportion of drugs distributed in Ukraine used to come from these countries, it could lead to a shortage of certain medicines, with potential negative repercussions to the health of Ukrainian citizens. Considering the above, the order No. 1801 of the Ministry of Health of Ukraine dated 05.08.2020 confirmed that the decision to ban the use of a medicinal product by terminating the validity of the registration certificate is not accepted if there are no analogues available in the Ukrainian market (zakon.rada.gov, 2022).

Another positive change in the regulation of the pharmaceutical sector set by the new Law on Medicinal Products, which will come into force 2.5 years after cancellation of the martial law, is the fact that the Ukrainian legislation on medicinal products will be adjusted to the requirements of the European Union (apteka, 2022b).

Because of the war, at the beginning of March, 2022 the Ministry of Health of Ukraine changed its approach to financing of the health care system. Every month, each hospital was guaranteed to receive 1/12 of the annual amount of funds from the National Health Service, irrespective of the number of services provided. It made it possible to maintain the system at a critical moment, to secure payment of salaries for medical workers as well as uninterrupted operation of hospitals. From 1 July 2022 in the regions that were not affected by hostilities, a standard payment system covering only medical services actually provided was reintroduced (kmu.gov, 2022).

Among the rescue projects successfully implemented by the Ministry of Health there are regular evacuation flights to secure the treatment of Ukrainians abroad in case their therapy is no more available in Ukraine. On average, there are about four of them per week. Since the beginning of the full-scale invasion, 1,274 Ukrainians have already been provided with medical assistance in leading clinics in 17 countries of the world (Skrypnyk, 2022d).

### Solutions provided by other stakeholders

Apart from the actions undertaken by the Ukrainian government, it is necessary to acknowledge the unprecedented sacrifice of both healthcare professionals and civil society (gov.ua, 2022; acmc.ua, 2022; Volontery, 2022). A special role in supporting maintenance of chronic therapies is played by pharmacists, especially in the zone of active hostilities (War: a pharmacist, 2022). In addition, pharmacists, as well as students and teachers of pharmaceutical universities implement various forms of volunteering, such as production of medicines for the military forces, participation in the collection of funds, items, food and medications, sorting of medical humanitarian aid, transportation of volunteer aid to the frontline area, *etc.* (new.meduniv, 2022a; new.meduniv, 2022b; new.meduniv, 2022c). The charity of both individual pharmacists and pharmacies, pharmaceutical enterprises and public pharmaceutical organizations, in particular the charity fund “All-Ukrainian Pharmaceutical Chamber - a single European family”, is also very important (Klimov, 2022). Volunteering and charity in Ukraine is closely related to the humanitarian mission of the global pharmaceutical community which is based on the principle of maximum effective use of donor funds, minimizing costs of logistics services for delivery of humanitarian goods directly to recipients, preventing commercialization of medicines and medical products received as humanitarian aid, and building of a transparent platform for collection, formation, delivery and reporting on distribution of humanitarian aid to a specific recipient (Half a year of war, 2022).

## Discussion

As a result of the Russian invasion, the Ukrainian healthcare system is facing multiple critical challenges. The war has caused massive internal and external migration of Ukrainian citizens and seriously destabilised the national economy. A great number of healthcare facilities have been destroyed, access to the others is often limited due to a lack of qualified medical staff. Not all drugs are fully available, even in the areas where no military attacks occur. The economic availability of the drugs is also profoundly affected as the cost of drugs has increased significantly and the average salaries have dropped down. The Government of Ukraine is trying to minimise the impact of the war on its healthcare system by adopting new legislation. However, the existing drug reimbursement program covers a limited number of medicines and health problems only. In the future, extending the list of diseases subject to drug reimbursement and introducing mandatory medical insurance can be effective solutions working towards improvements in long-term therapies in Ukraine.

Despite the hostilities are still in place, a look for future seems to be justified. This sort of approach now makes sense more than ever. As illustrated by the stress-test of healthcare system that COVID-19 pandemic conducted recently, many European countries are not well-prepared to maintain the continuity of long-term therapies in unfavourable conditions [ (Kardas et al., 2021)]. This is also a case of pharmaceutical service: a recent survey has shown that European hospital pharmacies are rather poorly prepared to emergencies and disasters (Schumacher et al., 2021). Fortunately, a crisis preparedness can be improved due to dedicated training (Schumacher et al., 2022).

Therefore, relevant actions needs to be taken in advance. In case of Ukraine, increasing the strength and resilience of the health and pharmaceutical system is advisable. Under the light of such principles, the National Council for the Restoration of Ukraine from consequences of the war proposes the project of the recovery plan for Ukraine. It includes planning to ensure the financial stability of the healthcare system, as well as restoration and transformation of the network of healthcare facilities. It pays special attention to strengthening preparedness for emergencies in the field of healthcare, reducing the dependence of the pharmaceutical sector on active pharmaceutical substances produced abroad, and last but not least, improving access to, and proper use of medicines (The project of the recovery plan of Ukraine, 2022).

These plans for post-war period are more than important. However, the armed conflict is not over now. In the unfavourable scenario set by it, multiple war-related factors create serious challenges to the maintenance of long-term therapies, and illustrate the reasons for which Ukraine needs urgent international support.

## Data Availability

The original contributions presented in the study are included in the article further inquiries can be directed to the corresponding author.
