# Supplementary material for: The impact of the war on maintenance of long-term therapies in Ukraine
Source: Front Pharmacol. 2022 Nov 24;13:1024046. doi: 10.3389/fphar.2022.1024046 (PMC9731218; doi:10.3389/fphar.2022.1024046)
Supplement: Supplementary file 1 [file Image1.pdf]

**Damaged**		**Completely ruined**
906	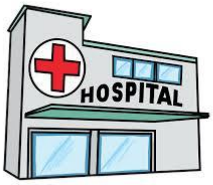	123
505	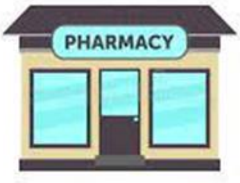	47
**Destroyed**		**Lost because of hostilities**
87	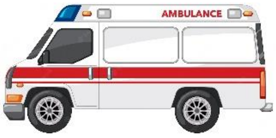	241
**Killed**	Among those not mobilized to the Armed Forces	**Injured**
18	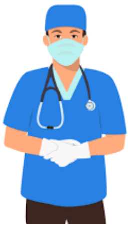	56
